# Biostimulant Response of Foliar Application of Rare Earth Elements on Physiology, Growth, and Yield of Rice

**DOI:** 10.3390/plants13111435

**Published:** 2024-05-22

**Authors:** Cynthia de Oliveira, Silvio Junio Ramos, Guilherme Soares Dinali, Teotonio Soares de Carvalho, Fábio Aurélio Dias Martins, Valdemar Faquin, Evaristo Mauro de Castro, Jorge Eduardo Souza Sarkis, José Oswaldo Siqueira, Luiz Roberto Guimarães Guilherme

**Affiliations:** 1 Departamento de Ciência do Solo, Universidade Federal de Lavras (UFLA), Lavras 37203-202, Brazil; cynthia.oliveira1@ufla.br (C.d.O.); teotonio.carvalho@ufla.br (T.S.d.C.); vafaquin@ufla.br (V.F.); jose.siqueira105@gmail.com (J.O.S.); 2 Instituto Tecnológico Vale—Desenvolvimento Sustentável, Rua Boaventura da Silva, 955, Belém 66055-090, Brazil; silvio.ramos@itv.org; 3 ICL, Avenida Doutora Ruth Cardoso, 8501, Butantã, São Paulo 04795-100, Brazil; 4 Empresa de Pesquisa Agropecuária de Minas Gerais (EPAMIG), Lavras 37200-000, Brazil; fabio.aurelio@epamig.br; 5 Departamento de Biologia, Universidade Federal de Lavras (UFLA), Lavras 37203-202, Brazil; emcastro@ufla.br; 6 Instituto de Pesquisas Energéticas e Nucleares (IPEN), Universidade de São Paulo (USP), Avenida Lineu Prestes, 2242, Cidade Universitária, São Paulo 05508-000, Brazil; jesarkis@ipen.br

**Keywords:** REE mix, photosynthetic performance, photochemical phase, chlorophyll *a*, photosynthetic rate, nutrient content, biostimulant dose

## Abstract

Rare earth elements (REEs) have been intentionally used in Chinese agriculture since the 1980s to improve crop yields. Around the world, REEs are also involuntarily applied to soils through phosphate fertilizers. These elements are known to alleviate damage in plants under abiotic stresses, yet there is no information on how these elements act in the physiology of plants. The REE mode of action falls within the scope of the hormesis effect, with low-dose stimulation and high-dose adverse reactions. This study aimed to verify how REEs affect rice plants’ physiology to test the threshold dose at which REEs could act as biostimulants in these plants. In experiment 1, 0.411 kg ha^−1^ (foliar application) of a mixture of REE (containing 41.38% Ce, 23.95% La, 13.58% Pr, and 4.32% Nd) was applied, as well as two products containing 41.38% Ce and 23.95% La separately. The characteristics of chlorophyll *a* fluorescence, gas exchanges, SPAD index, and biomass (pot conditions) were evaluated. For experiment 2, increasing rates of the REE mix (0, 0.1, 0.225, 0.5, and 1 kg ha^−1^) (field conditions) were used to study their effect on rice grain yield and nutrient concentration of rice leaves. Adding REEs to plants increased biomass production (23% with Ce, 31% with La, and 63% with REE Mix application) due to improved photosynthetic rate (8% with Ce, 15% with La, and 27% with REE mix), favored by the higher electronic flow (photosynthetic electron transport chain) (increase of 17%) and by the higher F_v_/F_m_ (increase of 14%) and quantum yield of photosystem II (increase of 20% with Ce and La, and 29% with REE Mix), as well as by increased stomatal conductance (increase of 36%) and SPAD index (increase of 10% with Ce, 12% with La, and 15% with REE mix). Moreover, adding REEs potentiated the photosynthetic process by increasing rice leaves’ N, Mg, K, and Mn concentrations (24–46%). The dose for the higher rice grain yield (an increase of 113%) was estimated for the REE mix at 0.72 kg ha^−1^.

## 1. Introduction

The rare earth elements (REEs) are a group of 15 elements belonging to the lanthanides with atomic numbers between Z = 57 and Z = 71 (i.e., lanthanum—La, cerium—Ce, praseodymium—Pr, neodymium—Nd, promethium—Pm, samarium—Sm, europium—Eu, gadolinium—Gd, terbium—Tb, dysprosium—Dy, holmium—Ho, erbium—Er, thulium—Tm, ytterbium—Yb, and lutetium—Lu), to which two more elements are added to form this homogenous group: scandium (Sc, Z = 21) and yttrium (Y, Z = 39) [1]. The term “earth” derives from the form in which their oxides are found, and the denomination “rare” is attributed to the difficulty of separating them, since they are usually found together in nature [2,3,4].

These elements have been used in Chinese agriculture since the 1980s to improve crop yields, mainly via the foliar application of fertilizers containing them [2,3,4,5,6,7,8]. Several results indicating yield increases of different crops treated with REEs have been reported in the literature, e.g., 5 to 15% in rice [9,10] and 12 to 24% in maize [5]. Additional data from Redling [6] have described yield increases for other crops, such as sugarcane and sugarbeet (8 to 20%); potato and Chinese cabbage (10 to 20%); cotton (5%); tobacco (16%); watermelon, banana, and orange (8%); and honey melon (114.4%).

These are evidence of the potential benefits of REEs for agriculture in other parts of the world, while this information is rarely found in the Americas.

Most commonly, crop yield increases have been often found with the use of foliar REE applications of a rare earth commercial formulation in China named “Changle” [2,6,11]. This fertilizer contains 25 to 28% of lanthanum oxide, 49 to 51% of cerium oxide, 5 to 6% of praesodymium oxide, 15 to 17% of neodymium oxide, and less than 1% of other rare earths [2]. Usually, these products contain the same four rare earth elements (Ce, La, Pr, and Nd), reflecting their high natural abundance in the environment [3].

Although REE-based products have been successfully marketed and used in China, their effects on plant physiology by promoting yield increases still need to be addressed. Many hypotheses have been proposed to explain such REE effects on plant growth and development. These statements range from metabolic and physiological modifications to genetic mechanisms, which are usually poorly documented.

Another dimension that should be considered in elucidating REE effects is their interaction with plant nutrients [4,12,13,14,15]. Indeed, it seems that REEs behave in plants with a hormesis effect (biphasic dose response), i.e., with a low-dose stimulation (beneficial stress/priming effect) and high-dose inhibitory/adverse reactions [16]. Therefore, biostimulant doses of these elements must be found for each species.

In order to understand the function of REE in plants, it is necessary to evaluate the effects that these elements cause on the physiological, metabolic, and growth characteristics that are part of the formation of the harvest (which promote grain yield) [4].

Under these circumstances, rice is a suitable cereal crop for studying whether REE is involved in photosynthesis and increased grain yield, since this crop has shown consistent response to REE application [12,17].

Rice was chosen for this study because it is a staple food for more than half of the world’s population and is grown in more than 100 countries, providing more than 20% of the calories consumed worldwide, mainly in Latin America, Asia, and India [18]. Besides this, rice is the most widely cultivated crop in China, where the great majority of the studies that prove the increase in grain yield from the foliar application of REEa are found [2,4].

Considering that our understanding of the biological role of REEs is still in its early stages [15,19], this study aimed to evaluate the effects of foliar application of REEs in the photochemical phase of photosynthesis in rice and its subsequent impact on plant nutritional status and development, as well as on crop yield. With that, it was hoped to unveil the role of REEs as prospective plant biostimulants, indicating the biostimulant dose for rice cultivation.

This manuscript is innovative for being the first to be carried out in pot and field conditions on tropical soils, where edaphoclimatic conditions are different from temperate conditions, considering rice cultivation, with a physiological approach and not just with agronomical endpoints considered. Studies carried out with plants in soils with different soil and climatic conditions are influenced by them and must be carried out to verify differences related to these contrasting conditions related to plant growth and development [20] (Moreira et al. 2019). The same applies to the need to evaluate physiological characteristics in an edaphoclimatic situation different from those already studied in a temperate climate [21] (de Souza et al. 2021).

## 2. Results

### 2.1. Experiment 1

#### 2.1.1. REE Concentrations in Shoots

The means of recovering REE contents in the certified material (BCR^®^ 670—Aquatic Plant (IRMM, Geel, Belgium) were as follows: Ce = 92.3%, La = 97.1%, Nd = 88.5%, and Pr = 83.1%. Such a recovery indicates reliable analytical accuracies for REE analysis.

REE concentrations were not detected in the shoots of the control plants with no REE application, while in the REE-treated plants, all applied elements were found (Table 1). The concentrations of Ce found in shoots in the Ce and REE mix treatments were 588.75 and 579.25 ng g^−1^, respectively, while La concentrations in shoots were 593.30 and 616.65 ng g^−1^ (Table 1). In the REE mix treatment, the Pr and Nd concentrations in the shoots were 137.38 and 378.44 ng g^−1^ (Table 1), respectively.

#### 2.1.2. Effect of REE on Characteristics of Fluorescence of Chlorophyll a, SPAD Index, Gas Exchanges, and Growth of Rice Plants

**Figure 1:**
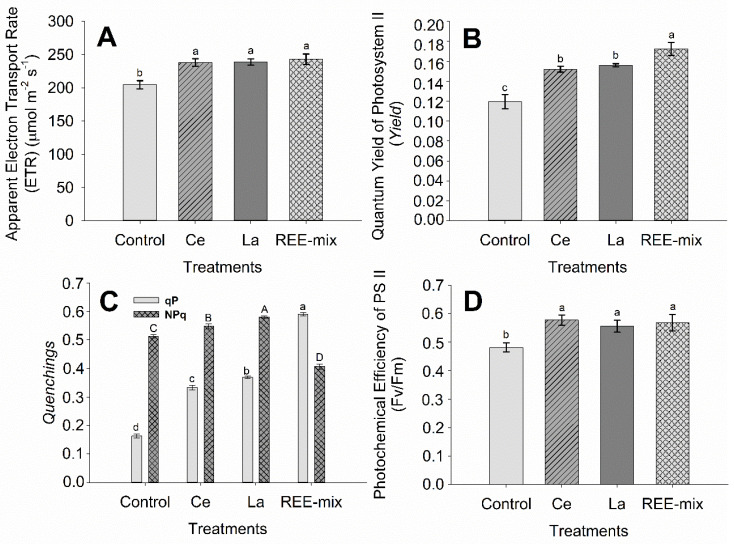
Fluorescence characteristics of chlorophyll *a* in rice plants grown in pots in full sunlight under foliar application of rare earth elements. (**A**): Apparent electron transport tate; (**B**): Quantum yield of photosystem II photochemistry; (**C**): Photochemical quenching and coefficient of non-photochemical extinction; and (**D**): Maximum photochemical efficiency of photosystem II (Fv/Fm Ratio). Graphical bars followed by the same letter did not differ significantly according to Tukey’s test; *p* < 0.05. Bars represent the mean standard error. (**C**): Photochemical quenching—lowercase letters; coefficient of non-photochemical extinction—capital letters.

In treatments receiving REEs, the apparent electron transport rate (ETR) increased in relation to the control (Figure 1A). This was observed when Ce and La were applied separately and as the REE mix (an increase of approximately 17%) (Figure 1A). The effective quantum yield of photosystem II photochemistry (ϕPSII) was very sensitive to the application of REEs, either when Ce and La treatments were applied separately (increase of approximately 20%), or—with a stronger effect—for the REE mix (an increase of approximately 29%) (Figure 1B).

For the quenchings, although the non-photochemical quenching (NPQ) increased with the Ce treatment and even more with the La treatment (an increase of approximately 2% and 12%, respectively), it decreased following the foliar application of the REE mix (decrease of approximately 23%) (Figure 1C).

The results of the photochemical quenching (qP), which increased with the application of Ce compared with the control treatment (increase of approximately 45%), were even higher with the application of La compared with the Ce treatment (increase of approximately 50%). The highest increase was observed with the application of the REE mix compared with all other treatments (an increase of approximately 69%) (Figure 1C).

Additionally, the maximum efficiency of photosystem II (F_v_/F_m_) increased significantly when plants were exposed to REEs via foliar application of both Ce and La separately and in the REE mix (increase of approximately 14%) (Figure 1D).

**Figure 2:**
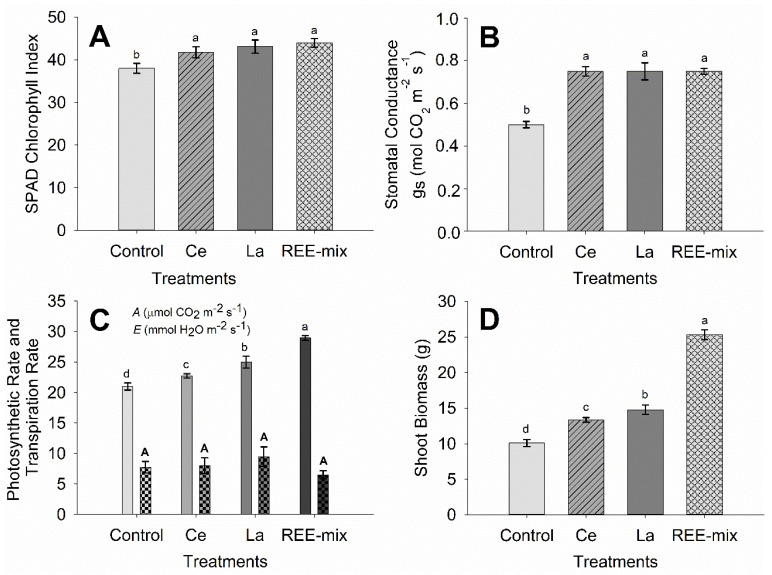
Physiological characteristics of the rice plants grown in pots in full sunlight under foliar application of rare earth elements. (**A**): Chlorophyll content (SPAD index), (**B**): Stomatal conductance, (**C**): Photosynthetic and transpiration rate; and (**D**): Shoot biomass. Graphical bars followed by the same letter did not differ significantly according to Tukey’s test; *p* < 0.05. Bars represent the mean standard error. (**C**): Photosynthetic rate—lowercase letters; transpiration rate—capital letters.

The SPAD index increased significantly when the plants were exposed to foliar application of REEs (an increase of approximately 10% with Ce, 12% with La, and 15% with REE mix application) (Figure 2A). Stomatal conductance (g_s_) also increased significantly when the plants were exposed to REEs, either when Ce and La were applied separately or when the REE mix was used in foliar applications (an increase of approximately 36%) (Figure 2B). The photosynthetic rate was intensified in the presence of REEs, mainly when the REE mix was applied (an increase of approximately 8% with Ce, 15% with La, and 27% with REE mix application) (Figure 2C). In turn, shoot biomass increased with the foliar application of Ce and La treatments separately, but even more when the REE mix was applied (an increase of approximately 23% with Ce, 31% with La, and 63% with REE mix application) (Figure 2D).

### 2.2. Experiment 2

#### 2.2.1. REE Concentrations in Leaves and Grains of Rice Plants

The quantification of REEs in plant material from experiment 2 was performed together with the analysis of plant material from experiment 1. Therefore, as reported for experiment 1, reliable analytical accuracy for REE quantification was also found for REE determinations in experiment 2.

**Figure 3:**
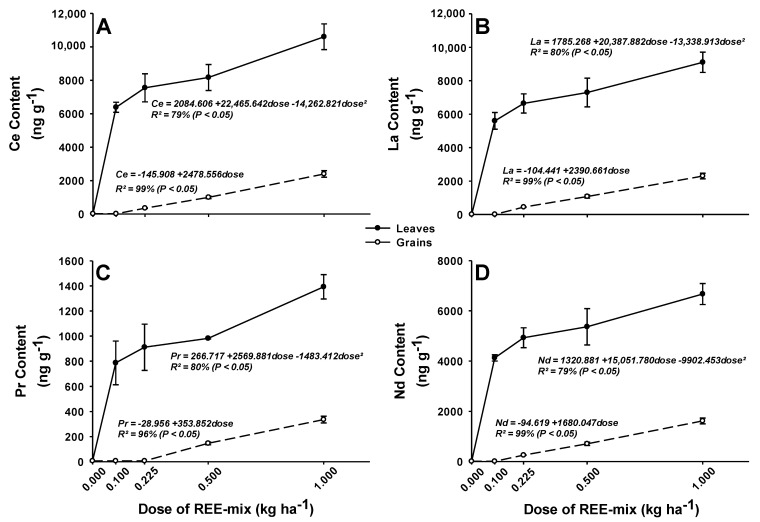
(**A**–**D**) Contents of rare earth elements in leaves and grains of rice. *p* < 0.05; bars represent the mean standard error.

In the control treatment without REE application, no REE was detected in the leaves (Figure 3A–D), indicating that the absorption of REE naturally occurring in the soil through the roots was negligible in the control treatment. A value corresponding to half the detection limit was used for each element to calculate the regression.

The application of increasing doses of REEs significantly increased their content in the leaves (Figure 3A–D). All analyzed REEs, both in leaves and grains, were linearly absorbed by rice plants (Figure 3A–D).

The concentrations of Ce found in the leaves varied from 6386.97 to 10,603.49 ng g*^−^*^1^ in the tested doses (Figure 3A) when the REE mix was applied. La concentrations in the leaves ranged from 5599.46 to 9104.11 ng g*^−^*^1^ (Figure 3B). Finally, for Pr and Nd, the concentrations found in the leaves ranged from 786.71 to 1392.94 ng Pr g*^−^*^1^ and from 4124.04 to 6671.25 ng Nd g*^−^*^1^ (Figure 3C,D).

The measurable concentrations of Ce found in the grains ranged from 355.25 to 2399.88 ng g*^−^*^1^ (Figure 3A) following the application of the REE mix. Quantifiable La concentrations in the grains ranged from 445.31 to 2302.711 ng g*^−^*^1^ (Figure 3B). Praseodymium was detected only in the grains of plants receiving the two highest doses of the REE mix (0.5 and 1.0 kg ha*^−^*^1^). None of the remaining REEs were detected when the REE mix was applied at the rate of 0.1 kg ha*^−^*^1^ (Figure 3C). However, for higher rates of the REE mix, the concentrations of these elements increased significantly in the grains (Figure 3A–D). For Pr, the quantifiable concentration ranged from 146.45 to 335.65 ng Pr g*^−^*^1^ (Figure 3C). Finally, the quantifiable concentrations for Nd varied from 253.27 to 1618.29 ng Nd g*^−^*^1^ (Figure 3D).

#### 2.2.2. Effect of REE Mix on Rice Grain Yield

**Figure 4:**
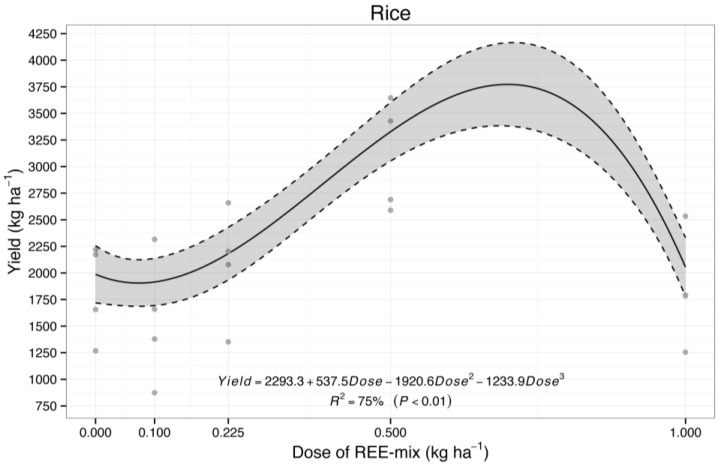
Rice grain yield treated with foliar application of an REE mix (Ce, La, Nd, and Pr).

For rice grain yield, a significant increase of 69% was observed when the REE mix was applied at a rate of 0.5 kg ha*^−^*^1^ (Figure 4). However, the applied modeling (cubic) (Figure 4) estimated that the highest yield would be obtained with 0.72 kg ha*^−^*^1^ of the REE mix, with an increase of 113% in rice grain yield.

At 0.225 kg ha*^−^*^1^ of the REE mix, a smaller increase of 14% in rice grain yield was observed (Figure 4).

#### 2.2.3. Effect of REE Mix on Mineral Rice Nutrition

To identify a possible interaction between the foliar absorption of REE and plant nutrients, the concentrations of macro- and micronutrients were determined in rice leaves and grains (Table 2). The REE mix applied at various doses affected nutrient concentrations in leaves and grains of rice differently (Table 2).

In the present study, magnesium (Mg) concentration in the leaves increased significantly at the doses of 0.225 and 0.5 kg ha^−1^ of the REE mix (an increase of approximately 20% with 0.225 kg ha^−1^, and 26% with 0.5 kg ha^−1^ REE mix application) (Table 2).

A significant increase in N concentration in rice leaves (Table 2) was observed up to the dose of 0.5 kg ha^−1^ of the REE mix (an increase of approximately 21%), for which there was the highest grain yield gain (69%) among all doses of the REE mix tested (Figure 2).

Although the concentrations of N and Mg increased significantly at 0.5 kg ha^−1^ of the REE mix in the leaves, the concentration of these nutrients in the grains did not differ statistically at any doses of the REE mix tested (Table 2).

Potassium (K) content also increased in rice leaves treated with 0.5 kg ha^−1^ of the REE mix (an increase of approximately 27%) (Table 2). Yet, when compared with the control treatment, the K content was reduced in the grains for the doses of 0.1, 0.5, and 1.0 kg ha^−1^ of the REE mix (a decrease of approximately 25% with 0.1 kg ha^−1^, 21% with 0.5 kg ha^−1^, and 27% with 1.0 kg ha^−1^ REE mix application) (Table 2).

For the doses of 0.1, 0.5, and 1.0 kg ha^−1^ of the REE mix, the concentration of manganese (Mn) in the leaves increased (an increase of approximately 27% with 0.1 kg ha^−1^, 46% with 0.5 kg ha^−1^, and 16% with 1.0 kg ha^−1^ REE mix application), whereas that of the grains decreased (a decrease of approximately 17% with 0.1 kg ha^−1^, 18% with 0.225 kg ha^−1^, 29% with 0.5 kg ha^−1^, and 48% with 1.0 kg ha^−1^ REE mix application) (Table 2).

Lastly, the boron (B) content increased in the doses of 0.1 and 0.225 kg ha^−1^ of the REE mix in the leaves (an increase of approximately 12% with 0.1 kg ha^−1^ and 0.225 kg ha^−1^ REE Mix application) and in the dose of 0.225 kg ha^−1^ of the REE mix in the grains (an increase of approximately 26%) (Table 2). Concerning iron (Fe), this nutrient had no changes in its concentrations in the leaves due to the increasing doses of the REE mix, but an increase in the dose of 0.225 kg ha^−1^ of the REE mix was observed for Fe in the grains (an increase of approximately 52%) (Table 2). The contents of phosphorus (P), calcium (Ca), sulfur (S), copper (Cu), and zinc (Zn) did not differ statistically in any of the tested doses of the REE mix in both leaves and grains (Table 2).

## 3. Discussion

### 3.1. Experiment 1

#### 3.1.1. REE Concentrations in Shoots

Means of recovery of REE contents in the certified material (BCR^®^ 670—Aquatic Plant (IRMM, Geel, Belgium) indicates reliable analytical accuracies for REE analysis.

All applied elements were found in the shoots of REE-treated plants, indicating that rice plants were able to absorb REE through foliar (leaf) application and exhibit the effects of these elements in their physiology, as described in the following sections.

In another study with the grass *Agrotis capillaris* [3], leaf concentrations of these elements were much smaller than those reported here. However, in a study with REEs in an agricultural soil of a rural area in Beijing (China) [22], the rice leaf concentration ranges for La, Ce, Pr, and Nd were similar to those found in our study (104.56–342.05, 212.22–632.88, 22.44–73.00, and 80.20–249.50 ng g^−1^), respectively. Similar values were observed by Sun et al. [23] when a commercial product named Nongle was sprayed on rice plants at the seeding stage. This fact confirms the ability of rice to absorb REEs via the leaves, which is species-dependent.

#### 3.1.2. Effect of REE on Characteristics of Fluorescence of Chlorophyll a, SPAD Index, Gas Exchanges, and Growth of Rice Plants

Before discussing data from the experiment under controlled conditions, it is noteworthy to mention that this study presents unique information concerning the effects of the foliar application of a mix of rare earth elements in plants (simulating REE fertilizers already used in Chinese agriculture) via analyses of the characteristics of the fluorescence of chlorophyll a, which has not yet been reported in the literature. This information is of great relevance for elucidating REEs’ effect on plants and explaining the increases in crop yield found in the presence of these elements.

In treatments receiving REEs, the apparent electron transport rate (ETR) increased in relation to the control, and this was observed when Ce and La were applied separately, as well as when used as the REE mix. This indicates that the electronic flux in the photochemistry phase increased in the presence of REEs, creating conditions for a more significant generation of energy (ATP) and reducing power (NADPH) for the biochemical phase of photosynthesis, potentiating this process. The effective quantum yield of photosystem II photochemistry (ϕPSII) was very sensitive to the application of REEs, either when Ce and La treatments were applied separately or—with a more substantial effect—for the REE mix. This suggests that in the presence of REEs, additional light energy absorbed by chlorophyll associated with PSII is available for use in photochemistry to generate more energy and reduce power for the photosynthetic process in the biochemical phase [24]. The fact that this effect was more pronounced when the REE mix was applied indicates the synergistic or complementary effect of these elements. Gong et al. [25] also observed different patterns between REE single and mixture applications. However, in a study with different concentrations of nano terbium (Tb) applications [26], it was observed that chlorophyll fluorescence (F_v_/F_m_ and F_v_/F_o_) was suppressed under 250–500 mg L^−1^ Tb, indicating toxicity. This adversely affected the trapped energy by the active reaction center of photosystem II (PSII) and led to an accumulation of inactive reaction centers, thus lowering the detected level of electron transport from photosystem II (PSII) to photosystem I (PSI).

For the quenchings, although the non-photochemical quenching (NPQ) increased with the Ce treatment and even more with the La treatment, it decreased following the foliar application of the REE mix. These data indicate that more energy flows into the photosynthesis by the application of the REE mix. Even though the NPQ was lower in the presence of the REE mix than in the control treatment, it was also enough to protect the PSII, since there was no indication of structural damage in PSII.

Concerning the photochemical quenching (qP), the increase provided by the application of Ce in relation to the control treatment, which was even higher with the application of La in relation to the Ce treatment, as well as upon the application of the REE mix in relation to all other treatments, promoted greater energy for the photochemical process.

Additionally, the maximum efficiency of photosystem II (F_v_/F_m_) increased significantly when the plants were exposed to REEs via foliar application of both Ce and La separately, as well as in the REE mix, and this indicates a great photosynthetic performance of rice due to REE foliar applications. The observed increases in ϕPSII, ETR, qP, and F_v_/F_m_ in the presence of REEs are strong evidence that these elements potentiate the photochemical phase of photosynthesis, increasing the energy (ATP) and reducing power (NADPH) generation for the biochemical phase [27], which may increase photosynthesis in rice plants.

An increase in the F_v_/F_m_ ratio was found by Wu et al. [28] in rice plants receiving foliar application of Ce (10 μM) in the presence of cadmium contamination. In a study with *Pseudostellaria heterophylla*, selected chlorophyll fluorescence parameters, e.g., F_v_/F_m_, ϕPSII, and ETR, decreased under perchlorate stress, but under appropriate La content (0.1 and 0.5 mg L^−1^ La^3+^), this decrease was alleviated [29]. In a work with *Dicranopteris dichotoma* grown in an REE mining area (i.e., under high REE native concentrations), increased efficiency of PSII and electron transport rate at low light intensities was reported [30]. Also, in a study with rice chloroplasts, changes in photosynthesis were observed due to an increase in Mg^2+^-ATPase activity in leaves of rice seedlings, as well as in rice plants in the booting and grain filling stages (in different amplitudes) at pH 4.5, under application of 0.08 mM of La, compared to the control treatment [31]. Indeed, the authors of the aforementioned study reported that the transcription level of chloroplast ATPase subunits α, β, ε, I, III, and IV increased [31], thereby improving the function of ATPase in producing ATP in the photochemical phase. Thus, REEs can increase the effectiveness of the electronic photochemical flux as observed in the present study, promoting ATPase performance and increasing the energy generation in the form of ATP for use in the biochemical phase of photosynthesis, which, in turn, potentiates the production of photoassimilates in rice. Hence, the increase of the electronic flow in the photosynthetic electron transport chain due to REE application can be directly related to the transcription of the ATPase subunits, as verified by Zhang et al. [31], as well as to conditioning a favorable pH for ATPase functioning in rice chloroplasts, since they act directly in the increase of electronic flux coupled to the translocation of protons, which create the transmembrane proton motive force, essential to ATP synthesis by ATP synthase.

In addition, REEs also act on the photosynthetic electron transport chain by increasing the levels of nutrients necessary for the electronic flow in the chloroplasts, for instance, manganese (Mn). Since Mn is required as an essential cofactor in the process of water oxidation and O_2_ generation, being associated explicitly with oxygen release complexes, it potentiates the photochemical phase of photosynthesis [32]. Given this, the increased electron transport rate observed in the presence of REEs, especially for the REE mix, may also be related to increased Mn concentrations in rice leaves, potentiating water oxidation and, consequently, the electron transport rate.

Moreover, Fa-Shui et al. [33] reported that La accelerated the transformation of light energy into electric energy, also increasing the electron transport of PSII, water photolysis, and oxygen evolution in spinach, which indicates that REEs can act in several regulation mechanisms of the photochemical phase.

The SPAD index increased significantly when the plants were exposed to foliar application of REEs, and a higher content of photosynthetic pigments means a greater photosynthetic capacity, which was observed in the presence of REEs. Thus, these elements can act as biostimulants of chlorophyll biosynthesis. Zhou et al. [34] applied 20 μM of Ce in a nutrient solution for maize growing under magnesium deficiency conditions. They reported that Ce prevented the inhibition of chlorophyll synthesis, also improving light absorption and transformation of energy, the evolution of oxygen, the photophosphorylation activity, and its coupling factor Ca^2+^-ATPase. In *Arabidopsis thaliana* cultivated in tissue culture, an increase in chlorophyll content has also been reported under Ce application (0.5 μM) [35]. On the other hand, Moreira [36] observed adverse effects of increasing Ce concentrations on the SPAD index in various plants (*Raphanus sativus*, *Helianthus annuus*, *Glycine max*, *Oryza sativa*, *Triticum aestivum*, *Zea mays*, and *Sorghum bicolor*). However, such effects varied among different plant species and soil types. In the aforementioned study, a decrease in the SPAD index in rice plants was observed only when the dose of 1206 mg kg^−1^ was applied in the soil [36].

Stomatal conductance (*g_s_*) also increased significantly when the plants were exposed to REEs, either when Ce and La were applied separately or when the REE mix was used in foliar application. Higher values of *g_s_* indicate higher CO_2_ input from the environment to the plant by the stomata, which results in greater carbon assimilation by photosynthesis. Consequently, the photosynthetic rate increased when Ce and La were applied separately, but even more so when the REE mix was applied. Overall, as a result of the photochemical phase being potentiated, which increases energy (ATP) and reduces power (NADPH) generation in the biochemical phase, and due to an increase observed in the chlorophyll and stomatal conductance, which increases CO_2_ input via stomata, also increasing carbon assimilation, we noted that the photosynthetic rate was intensified in the presence of REEs, mainly when the REE mix was applied.

An increase in the photosynthetic rate and stomatal conductance in rice growing in a nutrient solution with 81.6 μM of La [37] was attributed to the biostimulating effect of this REE on gaseous exchanges in rice. However, in the present study, the transpiration rate also was not responsive to the REE treatments. Maksimović et al. [38] observed that for maize plants growing in a nutrient solution, REE application decreased the transpiration rate, negatively affecting the absorption of nutrients by roots, which is governed by the transpiratory flow. Therefore, the maintenance of the transpiration rate after the application of REEs in rice, in the present study, indicates no negative effect of REEs on the absorption of nutrients by the plants, which is dependent on the transpiration flux. Salgado et al. [39] verified that adding Ce^3+^ to the nutrient solution would promote the growth of common bean, with an increase in photosynthesis rate, chlorophyll content, and water use efficiency under water stress.

In turn, shoot biomass increased with the foliar application of the Ce and La treatments separately, but even more so when the REE mix was applied. This resulted from an increase in the photosynthetic rate in the presence of REEs, mainly when the REE mix was used, as higher photosynthetic rates result in greater production of photoassimilates for plant growth and development. For soybean grown in the nutrient solution, Oliveira et al. [40] observed greater total chlorophyll content and an increase of 23% in the photosynthetic rate at the lowest concentrations of La applied (5 and 10 μM), resulting in a slight rise in root and shoot growth biomass. Hu et al. [41] and Guo et al. [42] also found increases in rice, maize, and sorghum biomasses after adding La or all REE, respectively. For *Triticum aestivum*, Shtangeeva et al. [43] verified, in soil-pot conditions, improved growth of roots and leaves with better architecture and structure. For *Triticum aestivum* and Secale cereal, soil application of Ce and Eu increased plant biomass [44]. Lastly, in tissue culture, *Tetrastigma hemsleyanum* also showed increased biomass after Ce application [45].

Shreds of evidence from the abovementioned data reveal that REEs could be considered a biostimulant for plant growth. Likewise, our data have shown that the REE mix stimulated the photochemical phase, promoting increases in chlorophyll (SPAD index) and stomatal conductance, thereby leading to a higher photosynthetic rate. This may result in the enhanced growth of rice plants, which may lead to higher grain yields. To validate these REE effects, a field trial was conducted, and the results are discussed next.

### 3.2. Experiment 2

#### 3.2.1. REE Concentrations in Leaves and Grains of Rice Plants

In the control treatment without REE application, no REE was detected in the leaves, indicating that the absorption of REEs naturally occurring in the soil through the roots was negligible in the control treatment. Again, a value corresponding to half the detection limit was used for each element to calculate the regression.

The application of increasing doses of REEs significantly increased their content in the leaves. This indicates that rice absorbs the REEs applied through the leaf, metabolizing them in their internal structures.

All analyzed REEs, both in leaves and grains, were linearly absorbed by rice plants. This indicates no saturation of the REE absorption sites at any of the doses tested. Therefore, if a more significant amount of REE was applied, it would still be absorbed.

The concentrations of Ce found in the leaves varied from 6386.97 to 10,603.49 ng g*^−^*^1^ in the tested doses when the REE mix was applied. La concentrations in the leaves ranged from 5599.46 to 9104.11 ng g*^−^*^1^. Finally, for Pr and Nd, the concentrations found in the leaves ranged from 786.71 to 1392.94 ng Pr g*^−^*^1^ and 4124.04 to 6671.25 ng Nd g*^−^*^1^. Ramirez-Olvera et al. [12] found that approximately 4700 ng g*^−^*^1^ Ce in rice shoots under 100 μM Ce were added to the nutrient solution for 28 d. Tyler [3] reported concentrations of 150, 110, 17, and 91 ng g*^−^*^1^ of Ce, La, Pr, and Nd in Agrotis capillaris leaves when REEs were added via soil applications. For rice, the leaves were reported to have 104.56–342.05, 212.22–632.88, 22.44–73, and 80.20–249.50 ng g*^−^*^1^ of La, Ce, Pr, and Nd, respectively, when REEs were soil-applied in a rural region of Beijing, China [22]. The REE concentrations found in the present study were considerably higher than in those previously described, probably because, in our study, they were directly applied to the leaves without risk of retention in the apoplastic barriers of the root or of being adsorbed or leached in the soil.

The fact that praseodymium was detected only in the grains of plants receiving the two highest doses of the REE mix (0.5 and 1.0 kg ha*^−^*^1^) was possibly because this element was present at the smallest proportion in the REE mix. Similarly, none of the remaining REEs were detected when the REE mix was applied at the rate of 0.1 kg ha*^−^*^1^, indicating little, if any, translocation to the grains of the REEs applied to the leaves. However, for higher rates of the REE mix, the concentrations of these elements increased significantly in the grains, indicating absorption and translocation to the grains of the REEs applied to the leaves.

The values reported here are higher than those found by Redling [6] in rice grains, for which only Y, La, Ce, Pr, and Nd could be determined quantitatively with values ranging from <0.78 to 5.58 ng g*^−^*^1^, whereas other REEs were close to or below the limit of detection. In the study conducted by Li et al. [22] with a soil located in a rural area of Beijing (China), the ranges of values of La, Ce, Pr, and Nd in rice grains were 1–6, 1.77–11.47, <1.26–1.97, and 0.85–5.27 ng g*^−^*^1^, respectively. Due to the apoplastic barriers present in the roots and stems, when present in the soil, REEs are retained more in these organs, and as a consequence, a lower content of these elements translocates to the grains. Indeed, Sun et al. [23] showed that higher contents of REEs move to the grains when these elements are applied in the leaves due to their lower retention by apoplastic barriers in the plant.

#### 3.2.2. Effect of REE Mix on Rice Grain Yield

For rice grain yield, a significant increase (69%), observed when the REE mix was applied at a rate of 0.5 kg ha*^−^*^1^, agrees with the positive effects of the REE mix on plant physiology and growth observed in experiment 1, i.e., the presence of REE—and mainly after the application of the REE mix—resulted in higher apparent electron transport rate, quantum yield of PS II photochemistry, photochemical quenching, maximum efficiency of PS II (Fv/Fm), and chlorophyll content, which all contributed to increased photosynthetic rate and biomass production.

At 0.225 kg ha*^−^*^1^ of the REE mix, a smaller increase of 14% in rice grain yield was observed. A rise of 7% in rice grain yield was reported by Wan [46], and additional studies with rice have also reported improvements of 5–10.3% [10] and 5–15% [9] in rice yield following the foliar application of REEs. Moreover, Xie et al. [47] reported that La at 0.05 mg L^−1^ to 1.5 mg L^−1^ stimulated rice yield gains of up to 72%.

Several mechanisms for these observed crop yield increases due to REE foliar application have been proposed at the metabolic, structural, and cytogenetic levels [4,15,40]. They include increased nutrient absorption and increased nitrogen fixation, as well as positive effects on biomass production by inducing higher photosynthetic rates resulting from increases in the electron transport rate in the photochemical phase of photosynthesis [2,3,29,48], as observed in experiment 1.

#### 3.2.3. Effect of REE Mix on Mineral Rice Nutrition

To identify a possible interaction between the foliar absorption of REEs and plant nutrients, the concentrations of macro- and micronutrients were determined in rice leaves and grains. The REE mix applied at various doses affected nutrient concentration in the leaves and grains of rice differently. The effects of REEs on plant mineral nutrition are diverse, which agrees with data reported in several studies describing either synergisms or antagonisms between the different plant nutrients and REEs [4,15]. Such effects also depend on the plant growing media and the method of REE application (e.g., foliar or soil application). In a study with soil application of REE under pot conditions, Liu et al. [17] found that La positively affected the concentration of Mg and Mn and negatively affected that of Ca in the roots. Using rice in tissue culture, Liu et al. [49] observed that the uptake of K and Ca in roots and shoots was positively affected by exposure to Ce up to 0.1 mM. Conversely, such effects were not found by Ramirez-Olvera et al. [12] in an experiment under controlled conditions (nutrient solution). Under field conditions, Ribeiro et al. [50] verified that foliar application of an REE mix (Ce, La, Pr, and Nd) did not affect the nutrient contents of leaves and grains of soybean and maize. These results demonstrate that REEs interact with other elements not only in the organs in which they are applied in the plants but also in different organs. In the case of foliar applications (e.g., the present study), foliar nutrition—and the factors governing it—will also affect the nutrition of the grains to be formed. However, Agathokleous et al. [51] described that REEs, such as Ce, can change the uptake and level in tissues of several micro- and macronutrients*,* depending on the dose, due to the hormesis effect observed in these elements.

In the present study, Mg concentration in the leaves increased significantly at the doses of 0.255 and 0.5 kg ha^−1^ of the REE mix. Such increases allowed a higher level of cellular Mg to be available for greater chlorophyll biosynthesis. In turn, a possible increase in chlorophyll concentration may have contributed to the improved photosynthetic rate, thereby providing conditions for the 14 and 69% increases in rice grain yield found at the doses of 0.225 and 0.5 kg ha^−1^ of the REE mix, respectively. Oliveira et al. [40] observed greater Mg content in the shoots of soybean plants exposed to La in nutrient solutions, reporting an increase in the chlorophyll content and the photosynthetic rate, up to 40 and 20 μM La, respectively. Ultimately, the observed increases in Mg content resulted in grain yield gains via an increased photosynthetic rate.

A significant increase in N concentration in rice leaves was observed up to the dose of 0.5 kg ha^−1^ of the REE mix, for which there was the highest grain yield gain (69%) among all doses of the REE mix tested. Besides constituting cellular components such as amino acids and nucleic acids, N—a vital component of the chlorophyll molecule—is the element required in greater quantity by plants for their development. For this reason, the higher N concentration observed in the leaves of 0.5 kg ha^−1^ of the REE mix contributed to the 69% increase in rice grain yield found at this dose of the REE mix applied. Makino [52], studying the photosynthetic performance and grain yield of rice in relation to N use, reported that increases in cereal yield depended on large inputs of N fertilizer. Accordingly, a greater availability of foliar nitrogen can promote increases in rice grain yield.

Although the concentrations of N and Mg increased significantly in the dose of 0.5 kg ha^−1^ of the REE mix in the leaves, the concentration of these nutrients in the grains did not differ statistically at any doses of the REE mix tested. However, the increase in these nutrients in the leaf, which is the photosynthetic organ, is directly linked to the production of grains [52]. As the chlorophyll molecule, which contains Mg and N, is essential for the production of photoassimilates, these nutrients are indispensable for the formation of photosynthates mobilized for the formation and filling of the grains. In addition, Mg is required for the activation of essential enzymes of the Calvin cycle (e.g., ribulose-1,5-bisphosphate carboxylase/oxygenase, fructose-1,6-bisphosphatase, sedoheptulose-1,7-bisphosphatase, and phosphoribulokinase) [53,54]. The N cycle in plants is also responsible for supplying intermediates of the plant carbon cycle through anaplerotic reactions [55]. Thus, the increase in the concentration of these two elements in the leaves is fundamental to the rise in grain yield. To sum up, the highest grain yield found in the 0.5 kg ha^−1^ dose of the REE mix may also be due to the higher concentration of Mg and N available for greater chlorophyll biosynthesis, higher activation of key enzymes of the carbon metabolism, and production of carbon cycle intermediates through interaction with the nitrogen cycle.

Additional data from the literature reported increases in the concentration of Mg in maize and mungbean at 5 μM of La, respectively [56,57]. Also, Hu et al. [2] and Wei et al. [58] have shown that foliar application of Nd promoted the redistribution of N in rapeseed towards plant parts treated with this REE. Moreover, according to Hu et al. [2] and Jie et al. [59], the application of REEs increased the efficiency of N in wheat.

Potassium (K) content also increased in rice leaves treated with 0.5 kg ha^−1^ of the REE mix. The role of K in governing cellular turgor, especially in the regulation of guard cells, and its protagonist as an enzymatic cofactor in numerous metabolic processes of carbon and nitrogen cycles in plants [60], have direct action in the final production of photosynthates. Therefore, a higher availability of K for these metabolic functions allows for a greater production of photoassimilates and, consequently, a higher yield of grains. Yet, the content of K was reduced in the grains for the doses of 0.1, 0.5, and 1.0 kg ha^−1^ of the REE mix compared with the control treatment. This may have occurred because K remained in a higher concentration in the leaves, being less translocated to the grains. Oliveira et al. [40] observed an increase in the K content of roots and shoots for soybeans, with a rise in the La concentration applied. In contrast, in rice shoots, decreases in K content were observed following the application of increasing doses of Ce [49]. These facts indicate that REEs interact differently with plant nutrients because of variations concerning crop differences, methods, and forms of REE application (e.g., single or mixed REE formulations, as in the present study), as well as different amounts applied.

The concentration of manganese (Mn) in the leaves increased, whereas that of the grains decreased forthe doses of 0.5 and 1.0 kg ha^−1^ of the REE mix. It is possible that the increased manganese (Mn) concentration in these doses of REEs happened in the leaves because this organ needs to perform its function in metabolic processes that culminate in the formation of reserves to be mobilized for the formation and filling of the grains. Since Mn is required as an essential cofactor in water oxidation and O_2_ generation, being associated explicitly with oxygen release complexes, it has activity in the photochemical phase of photosynthesis [32]. Therefore, the electron transport rate increase observed in experiment 1 in the presence of REE and especially in the presence of the REE mix may be related to the rise of the Mn content in rice leaves, potentiating the oxidation of water and, consequently, the rate of transport of electrons. In a study with soybean, Oliveira et al. [40] observed for both roots and shoots that exposure to increasing concentrations of La (up to 10 μM of La in solution) resulted in increased contents of Mn as well as an increased photosynthetic rate. Conversely, in a study with rice exposed to Ce in the nutrient solution, Ramirez-Olvera [12] found that the concentration of Mn in roots and shoots was not significantly affected by the application of Ce compared with the control treatment.

The contents of P, Ca, S, Cu, and Zn did not differ statistically in any of the tested doses of the REE mix in both leaves and grains. Thus, the function of these elements was maintained in the presence of REEs due to the maintenance of their contents to levels comparable to those observed for the control treatment.

Considering the aforementioned results found in this work for the interaction between the REEs—applied as an REE mix—and the nutrients in rice plants, as well as the increase in grain yield in rice, the dose of 0.5 kg ha^−1^ of the REE mix can be indicated as the ideal concentration for providing the beneficial effect of REEs. Indeed, this dose brings together the greatest number of beneficial interactions derived from the application of the REE mix, i.e., it increases nutrients in rice, culminating with an increase of 69% in grain yield. Finally, considering the definition of biostimulants—i.e., all substances or materials, except for nutrients and pesticides, that applied to the plant have the capacity to beneficially modify its growth [61]—or findings suggested that REEs applied at the dose of 0.5 kg ha^−1^ of the REE mix can be considered biostimulants in terms of the mineral nutrition of rice plants, with consequent increases of grain yield.

The recommended amount to be applied of these elements as biostimulants of plant growth and crop yield is small, as seen in our study. The highest dose studied (1 kg ha^−1^) of the REE mix is equivalent to an application of 2.069 mg kg^−1^ of Ce, 1.198 mg kg^−1^ of La, 0.216 mg kg^−1^ of Pr, and 0.678 mg kg^−1^ of Nd if we consider the arable layer of 0.20 m of soil. The dose of 0.5 kg ha^−1^ of the REE mix that was indicated as the ideal concentration for providing the beneficial effect of REE in this study provides 1.035 mg kg−^1^ of Ce, 0.599 mg kg^−1^ of La, 0.108 mg kg^−1^ of Pr, and 0.339 mg kg^−1^ of Nd, considering the arable layer of 0.20 m of soil.

The study of Moreira et al. [20] with eight crop species (corn, sorghum, rice, wheat, soybeans, sunflower, radish, and beans) exposed to a Ce concentration gradient in two typical tropical soils (Oxisol and Inceptsol) revealed that REE cerium (which occurs in major concentrations in our study and in the environment) is not extremely hazardous to terrestrial plants. These authors supported this idea by the results observed by their risk assessment as follows: hazardous concentration, which is the Ce concentration that would cause risk to 5% or less of all plant species tested (HC_5_) was 281.6 mg Ce kg^−1^, 136-fold more than the highest dose of Ce applied in our study, and 272-fold more than the dose indicated as adequate of Ce in this study.

Moreira et al. [20] observed that rice showed higher EC_50_ values (the effective concentration of Ce resulting in 50% inhibition derived by concentration response), regardless of the soil type (Oxisol or Inceptisol), and was the most tolerant species for Ce. For shoot dry matter, the EC_50_ values were 691.8 and 1325.2 for Oxisol and Inceptisol, respectively. For the germination speed index, the predicted value exceeded the range of concentrations evaluated in the experiment (cerium concentrations used: 0 (control), 50, 85, 144.5, 245.7, 417.6, 709.9, 1206.9, and 2051.7 mg Ce kg^−1^) for Oxisol, and the effect of Ce could not be estimated for Inceptisol. No observed effect concentration (NOEC) for rice in the study of Moreira et al. [20] was 709.9 mg Ce kg^−1^ in Oxisol and Inceptisol, and the lowest observed effect concentration (LOEC) was 1206.9 mg Ce kg^−1^.

A study with lanthanum (the second element that occurs in major concentration in our study and in the environment) showed a value of HC_5_ of 49 mg kg^−1^ dry soil when it was derived using EC_10_ data from soil invertebrates, bacteria, and plants [62]. This value is 81-fold more than the value indicated for La in our study. Therefore, rare earth element concentrations utilized in our study were smaller than the concentrations that the studies of toxicology indicated as to the beginning of problems for primary organisms such as plants.

Furthermore, in general, these elements tend to be more retained in the leaves, and their concentrations decline in seeds and fruits due to apoplastic barriers and a low translocation to edible parts such as grains, as in the case of rice [2,4]. In this study, for example, the measurable concentrations of Ce, La, Pr, and Nd, respectively, found in the grains ranged from 355.25 to 2399.88 ng g^−1^ (Ce), from 445.31 to 2302.711 ng g^−1^ (La), from 146.45 to 335.65 ng g^−1^ (Pr), and from 253.27 to 1618.29 ng g^−1^ (Nd). Therefore, the values that the grains could reach for animals and humans are quite low. However, still, additional research is required about guidelines for recommended daily limits of REE content in foods.

## 4. Materials and Methods

### 4.1. Experiment 1—Pot Conditions

#### 4.1.1. Plant Material, Treatments, and Experimental Design

Rice plants (*Oryza sativa* L. cv BRSMG Caçula) [63] were grown under full sunlight conditions for 68 days (until the pre-flowering stage [63]) in 5 kg pots (20 cm high, 20 cm wide at the base, and 21 cm in diameter) filled with samples from the 0–20 cm surface layer of a *Latossolo Vermelho Distroférrico Típico* [64], corresponding to an Anionic Acrudox [65], whose chemical and physical attributes are presented in Table 1. Soil pH adjustment with lime and fertilization (planting and top-dressing fertilization) were performed as proposed by Malavolta et al. [66] as follows: 300 mg N dm^−3^; 300 mg P dm^−3^; 150 mg K dm^−3^ (N and K divided into one cultivation application and two coverage applications); 90 Ca mg Ca dm^−3^ (soil correction by liming); 30 mg Mg dm^−3^ (soil correction by liming); 50 mg S dm^−3^; 0.5 mg B dm^−3^; 1.5 mg Cu dm^−3^; 0,1 mg Mo dm^−3^, and 5 mg Zn dm^−3^. The Caçula rice cultivar was used because it is a reference cultivar used in much of the Brazilian rice breeding research and in several Brazilian states under farmers’ field conditions [63]. It was the most indicated cultivar in the Minas Gerais States, where experiments 1 and 2 [62] were conducted. The total cycle of this cultivar lasts an average of 100 to 110 days, with 50% flowering after 72 to 76 days [63].

The experiment followed a completely randomized design with four treatments (control, Ce, La, and REE mix) and five replicates. Each plot consisted of one pot with soil containing two rice plants. The four treatments consisted of foliar applications of the different solutions 56 days after sowing, i.e., at the booting stage [63]. To obtain the treatment corresponding to the REE mix, 411 g ha^−1^ (0.411 kg ha^−1^) of the pure salts for analysis (p.a.) of the main components of “Changle” were applied in proportion to their occurrence in this REE fertilizer, as described by Wen et al. [67] (41.38% Ce, 23.95% La, 13.58% Pr, and 4.32% Nd). In this application, the following values were applied: 170.07 g Ce ha^−1^, 98.43 g La ha^−1^, 17.76 g Pr ha^−1^, and 55.72 g Nd ha^−1^. These values per plant are as follows: 0.12 mg Ce plant^−1^, 0.07 mg La plant^−1^, 0.01 mg Pr plant^−1^, and 0.04 mg Nd plant^−1^ (considering 1,400,000 plants ha^−1^, since 70 plants per linear meter were considered, with spacing between rows in the field of 0.5 m and spacing between plants of 0.014 m. The same proportions of Ce (41.38%) and La (23.95%) were applied separately for the characterization of the Ce (170.07 g ha^−1^) and La (98.43 g ha^−1^) treatments, respectively. The solutions of the different treatments were diluted with distilled water and combined with an adjuvant (Assist ^®^ 756 g L^−1^ of mineral oil, 0.5% *v*/*v*) (BASF, Ludwigshafen, Germany). A dose equivalent to the manufacturer’s recommendation of 1 L ha^−1^ (0.875 mL per pot) was applied. Only distilled water and adjuvants were applied to characterize the control treatment (with no REE). The volume of syrup applied was 0.150 L plant^−1^. The sources of REEs were Ce(NO_3_)3.6H_2_O, La(NO_3_)3.6H_2_O, Pr(NO_3_)3.6H_2_O, and Nd(NO_3_)3.6H_2_O, respectively (Sigma—Aldrich, St. Louis, MO, USA). Since nutrient fertilization (planting and top-dressing fertilization) was made, as proposed by Malavolta et al. [66], with 300 mg N dm^−3^ soil, the supply of N to all treatments was standardized. As nitrogen is a macronutrient, the very low amount of N supplied via foliar application of REEs can be considered insignificant.

#### 4.1.2. Shoot Biomass and REE Concentrations

At harvest, the shoots were dried in a forced-air drying oven (SolidSteel 630 L, SolidSteel, Belo Horizonte, Minas Gerais, Brazil) at 60 °C until a constant mass was reached to obtain the dry mass. For the REE analyses, 0.5 g of shoot dry mass was ground in a Wiley mill, model R-TE-648 (Tecnal, Piracicaba, São Paulo, Brazil). Subsequently, these aliquots were digested in 5 mL of HNO_3_ p.a. using a microwave digestion oven, Mars 5 (CEM, Berkeley, California, USA), for 30 min at a pressure of 0.76 MPa. After digestion, the extract was filtered and its volume was completed to 10 mL with distilled water. The samples were digested according to the protocol of the United States Environmental Protection Agency method 3051 A [68]. A certified reference material (Aquatic Plant—BCR670^®^, Institute for Reference Materials and Measurements, IRMM, Geel, Belgium) was included for quality control. Blank and certified reference samples were also analyzed along with every digestion batch (n = 7). REE concentrations in the extracts were determined by inductively coupled plasma mass spectrometry (ICP-MS) (Model Nex ION 300D, PerkinElmer, Waltham, MA, USA).

#### 4.1.3. Photochemical Characteristics of Photosynthesis

At 68 days (pre-flowering stage) [63], two fully expanded leaves for each of the two plants of each replicate in each treatment were dark-adapted for 40 min for chlorophyll a fluorescence measurements, using a Mini-PAM chlorophyll fluorometer (Mini-PAM II, Walz, Effeltrich, Germany), with the aid of leaf clips (Walz, Effeltrich, Germany). With that, the maximal quantum yield of photosystem II photochemistry (Fv/Fm) was obtained. In the light, values of the effective quantum yield of PSII (ΦPSII), photochemical quenching (qP), nonphotochemical quenching (NPq), as well as apparent electron transport rate (ETR) were obtained by actinic light pulses applied through a fiber optics pointing at 60° on the leaf.

#### 4.1.4. SPAD Index

The chlorophyll meter SPAD 502 Plus (Konica Minolta Co., Ltd., Osaka, Japan) was used to obtain the SPAD index (Soil Plant Analysis Development Index) value on the last fully expanded leaf from rice for each of the two plants of each replicate of each treatment after 68 days (pre-flowering stage) [63]. For each SPAD evaluation, three measurements on the last fully expanded leaf were used, and the average of those measurements was used for analysis.

#### 4.1.5. Gas Exchange Analysis

Gas exchange characteristics were analyzed after 68 days (pre-flowering stage) [63] using an infrared gas exchange analyzer, IRGA (Li-6400 XT, Li-Cor, Lincoln, NE, USA). Stomatal conductance (*g_s_*), transpiration rate (*E*), and photosynthetic rate (*A*) were measured as follows: in the four replicates of each REE treatment, three fully expanded leaves were selected at 9 a.m., and the density of the photosynthetically active photon flux was fixed in the device chamber at 1000 μmol m^−2^ s^−1^. This evaluation was performed on each of the two plants of each replicate of each treatment.

#### 4.1.6. Statistical Analysis

All data were subjected to analysis of variance (ANOVA), and the means were compared by the Scott–Knott at a 0.05 significance level of probability using Sisvar 5.3 (Build 77) statistics software [69]. Graphs were made using Sigma Plot software (version 12.5, Systat Software, Chicago, IL, USA).

### 4.2. Experiment 2—Field Conditions

#### 4.2.1. Plant Material, Treatments, and Experimental Design

In the field experiment, rice (*Oryza sativa* L. cv Caçula) [63] plants were grown in a farm field of the Agricultural Research Enterprise of Minas Gerais (EPAMIG), Lambari, MG, Brazil (latitude: 21°58′10″ S; longitude: 45°22′00″ W and 896 above msl), until the end of the plant cycle (115 days) [63]. “This field experiment was conducted in the autumn season of 2015 (March to June 2015), when the local average temperature was 18.94 °C (with a minimum temperature of 8.2 °C and a maximum of 29.4 °C), and the total precipitation in this whole period was 334.5 mm.” The soil used for this experiment was a *Latossolo Vermelho Distrófico Típico* [64], corresponding to a Typic Hapludox [65], for which the results of chemical and physical analyses are given in Table 1. Before rice was planted, basal fertilization with NPK was performed at a rate of 300 kg ha^−1^ (formula 08-28-16). Top dressing fertilization with 250 kg ha^−1^ of NK (formula 20-00-20) was applied 36 days after planting (DAP). Experimental plots contained 10 rows of 5.0 m in length with an inter-row spacing of 0.20 m (total area of 12 m^2^), and the total useful area (effectively used for yield evaluation) was 4.8 m^2^. The experiment was set as a randomized complete block design with four replications. The treatments were characterized as follows: control (distilled water + adjuvant), 0.1, 0.225, 0.5, and 1 kg ha^−1^ of the REE mix used in experiment 1. In the application of 1 kg ha^−1^ of the REE mix, the following values were applied: 413.8 g Ce ha^−1^, 239.5 g La ha^−1^, 43.2 g Pr ha^−1^, and 135.58 g Nd ha^−1^. In the other doses, proportional values were used. The volume of syrup to application used was 300 L ha^−1^. All solutions containing REEs were diluted with distilled water and combined with an adjuvant (Assist^®^ 756 g L^−1^ of mineral oil, 0.5% *v*/*v*) (BASF, Ludwigshafen, Germany). A dose equivalent to the manufacturer’s recommendation of 1 L ha^−1^ was applied. The foliar application was made at the booting stage (at 56 days) [63]. Cultural practices, such as application of the herbicides ethoxysulfuron (81 g a. i ha^−1^) + metsulfurom methyl (2 g a.i ha^−1^) and the fungicide triciclazol (225 g a. i ha^−1^) were performed at 24 DAP and 73 DAP, respectively.

#### 4.2.2. REE Concentrations in Shoots and Grains

The analyses of REE concentrations in shoots and grains were performed as described in experiment 1.

#### 4.2.3. Grain Yield

To obtain the rice grain yield, the grains were harvested manually in the useful area and subsequently treated and cleaned using the stationary machine LCZX-50 (Zaccaria, Limeira, São Paulo, Brazil). They were then stored in a forced circulation air oven (SolidSteel 630 L, SolidSteel, Belo Horizonte, Minas Gerais, Brazil) at 60 °C and dried to 13% moisture. The grain yield (kg ha^−1^) was calculated based on the corrected grain weight (adjusted to 13% moisture) and the planted area of the plots.

#### 4.2.4. Nutrient Concentrations

For macro- and micronutrient analyses, dried tissue (500 mg) was weighed and digested with 4.0 mL of concentrated HNO_3_ ± 2.0 mL of concentrated HClO_4_ (Sigma–Aldrich, Saint Louis, MO, USA) at 120 ± 8 °C for 1 h and then at 220 ± 8 °C until HClO_4_ fumes were observed. Total Ca, Mg, K, Cu, Mn, Fe, and Zn concentrations in the samples were determined using the atomic absorption spectrophotometer AAnalyst 800 (PerkinElmer, San Jose, CA, USA); total S concentration was determined using turbidimetry of barium sulfate; and total P was determined using a spectrophotometer (UV/VIS EasyPlus, Mettler Toledo, Columbus, OH, USA) to measure the colorimetry of a phospho-molybdenum complex at 680 nm [66]. The total N concentration was determined using the Kjeldahl method, described by Bremner and Keeney [70].

#### 4.2.5. Statistical Analysis

Grain yield data were analyzed using polynomial regression in the R environment [71]. All variables were subjected to analysis of variance (ANOVA), and the means were compared by the Scott–Knott at a 0.05 significance level of probability using Sisvar 5.3 (Build 77) statistics software [67].

## 5. Conclusions

Adding REEs via foliar application, mainly in the form of an REE mix (Ce, La, Pr, and Nd), to rice plants increased photosynthesis. The observed growth benefits of REEs may be related to their physiological effects on higher electronic flow in the photosynthetic electron transport chain and the higher Fv/Fm and quantum yield of photosystem II, as well as increased stomatal conductance and the SPAD index.

The higher electronic flow, which contributed to the increase in the photosynthetic rate and biomass production observed in experiment 1 (with 411 g ha^−1^ of the REE mix), could be the reason for the greater grain yield observed for the REE mix rate of 0.5 kg ha^−1^ tested in experiment 2, as well as for the higher levels of N, Mg, K, and Mn found in the leaves of this treatment.

Thus, the dose of 0.5 kg REE mix ha^−1^ can be indicated as the ideal concentration for biostimulating rice crops because this concentration brings together the maximum number of beneficial interactions derived from the application of the REE mix, resulting in better nutrient concentrations in rice and culminating in a 69% grain yield gain. However, the greater biostimulant effect of the REE mix on the crop was estimated at the dose of 0.72 kg REE mix ha^−1^, with an estimated increase of 113% in rice grain yield.

To the best of our knowledge, our findings represent the most comprehensive study concerning the foliar application of REEs in rice, with the most significant grain yield increases observed so far.

In further studies with REEs applied to rice, it is necessary to evaluate the genetic and molecular mechanisms underlying the interactions between the macro- and micronutrients, particularly N, Mg, K, and Mn, mainly at the concentration of 0.5 kg h^−1^ of REE mix in the leaves, and to use two cultivation seasons.

## Figures and Tables

**Table 1:** Contents of rare earth elements in shoots of rice.

Treatments	Rare Earth Element			
	Ce	La	Pr	Nd
	ng g^−1^			
Control	n.d.	n.d.	n.d.	n.d.
Ce	588.75 ± 48.83	n.d.	n.d.	n.d.
La	n.d.	593.30 ± 30.57	n.d.	n.d.
REE mix	579.25 ± 15.92	616.65 ± 64.58	137.38 ± 10.37	378.44 ± 95.90

± Standard error of the mean; n.d. not detected.

**Table 2:** Nutrient concentrations in rice leaves and grains under the application of the REE mix.

Nutrients	Nutrient Concentration in Leaves for Each REE Mix Treatment (kg ha^−1^)				
	0	0.1	0.225	0.5	1.0
N (g kg^−1^ DW)	8.70 ± 0.3606 b	9.50 ± 0.2784 b	9.10 ± 0.3122 b	11.03 ± 0.4537 a	8.93 ± 0.2466 b
P (g kg^−1^ DW)	0.60 ± 0.0132 a	0.56 ± 0.0076 a	0.57 ± 0.0076 a	0.62 ± 0.0161 a	0.58 ± 0.0132 a
K (g kg^−1^ DW)	14.15 ± 0.4479 b	16.02 ± 0.3184 b	17.09 ± 0.6769 b	19.48 ± 0.2952 a	15.75 ± 0.7970 b
Ca (g kg^−1^ DW)	3.63 ± 0.1351 a	3.35 ± 0.0828 a	3.94 ± 0.0835 a	3.93 ± 0.2105 a	3.32 ± 0.1583 a
Mg (g kg^−1^ DW)	1.92 ± 0.0633 b	1.82 ± 0.1037 b	2.41 ± 0.1678 a	2.61 ± 0.1477 a	1.85 ± 0.1675 b
S (g kg^−1^ DW)	1.50 ± 0.0050 a	1.48 ± 0.0126 a	1.60 ± 0.0562 a	1.56 ± 0.0058 a	1.49 ± 0.0229 a
B (mg kg^−1^ DW)	6. 63 ± 0.4381 b	7.59 ± 0. 0000 a	7.59 ± 0.0000 a	5.87 ± 0.2850 b	6.82 ± 0.3320 b
Cu (mg kg^−1^ DW)	5.51 ± 0.8101 a	5.33 ± 0.0650 a	4.91 ± 0.3219 a	7.50 ± 0.6283 a	5.94 ± 0.2291 a
Fe (mg kg^−1^ DW)	607.95 ± 128.1468 a	394.81 ± 58.3299 a	298.48 ± 46.5421 a	250.89 ± 33.7862 a	486.72 ± 102.9976 a
Mn (mg kg^−1^ DW)	314.81 ± 7.7011 b	432.04 ± 18.5765 a	362.08 ± 17.7165 b	578.99 ± 39.7377 a	375.66 ± 33.0049 a
Zn (mg kg^−1^ DW)	41.87 ± 6.3462 a	53.56 ± 3.1036 a	54.59 ± 3.0711 a	61.33 ± 2.4874 a	42.97 ± 7.0213 a
Nutrients	Nutrient Concentration in Grains for Each REE Mix Treatment (kg ha^−1^)				
	0	0.1	0.225	0.5	1.0
N (g kg^−1^ DW)	16.73 ± 0.3547 a	16.23 ± 0.2754 a	15.17 ± 0.3253 a	16.30 ± 0.3122 a	16.17 ± 0.3055 a
P (g kg^−1^ DW)	2.94 ± 0.1207 a	2.67 ± 0.1156 a	2.50 ± 0.0501 a	2.45 ± 0.0076 a	2. 68 ± 0.0690 a
K (g kg^−1^ DW)	3.37 ± 0.2599 a	2.54 ± 0.0247 b	3.25 ± 0.0861 a	2.68 ± 0.1147 b	2.47 ± 0.1476 b
Ca (g kg^−1^ DW)	0.62 ± 0.0568 a	0.51 ± 0.0328 a	0.61 ± 0.0132 a	0.52 ± 0.0577 a	0.56 ± 0.1660 a
Mg (g kg^−1^ DW)	1.35 ± 0.0551 a	1.20 ± 0.0563 a	1.15 ± 0.016 a	1.12 ± 0.369 a	1.13 ± 0.0723 a
S (g kg^−1^ DW)	1.22 ± 0.0029 a	1.20 ± 0.0275 a	1.19 ± 0.0132 a	1.21 ± 0.0275 a	1.25 ± 0.0050 a
B (mg kg^−1^ DW)	2.71 ± 0.0000 b	1.25 ± 0.1559 d	3.64 ± 0.1617 a	3.08 ± 0.1617 b	2.16 ± 0.0000 c
Cu (mg kg^−1^ DW)	5.71 ± 0.0247 a	5.79 ± 0.2499 a	5.82 ± 0.1291 a	5.38 ± 0.1239 a	5.51 ± 0.0321 a
Fe (mg kg^−1^ DW)	763.95 ± 147.1187 b	173.68 ± 29.4010 b	1594.70 ± 385.5715 a	158.23 ± 8.4310 b	111.53 ± 24.8830 b
Mn (mg kg^−1^ DW)	139.20 ± 7.0452 a	115.19 ± 4.6474 b	113.78 ± 5.3530 b	99.32 ± 4.4464 c	78.22 ± 9.5380 c
Zn (mg kg^−1^ DW)	36.36 ± 0.2397 a	33.71 ± 0.2757 a	34.68 ± 0.7671 a	36.04 ± 1.5273 a	33.95 ± 0.2875 a

Different letters in the rows indicate significant differences (*p* < 0.05) according to the Scott–Knott test. DW: dry weight; ± standard error of the mean; n = 4.

## Data Availability

Data are contained within the article.
